# The relationship between medical students’ attitudes toward artificial intelligence and their personality traits: a multicenter study in China

**DOI:** 10.3389/fpubh.2026.1749279

**Published:** 2026-02-04

**Authors:** Jinxin Qi, Yuxiao Zeng, Hao Wang, Yuchu Xiang, Zitong Fang, Yinghan Zhang, Tingting Bao, Shuyu Yan, Lian Liu, Yaoxi Su, Xian Jiang, Siliang Chen

**Affiliations:** 1 Department of Dermatology and Venereology, West China Hospital, Sichuan University, Chengdu, China; 2 Laboratory of Dermatology, Clinical Institute of Inflammation and Immunology, Frontiers Science Center for Disease-related Molecular Network, State Key Laboratory, Chengdu, China; 3 West China School of Public Health, West China Medical Center, Sichuan University, Chengdu, China; 4 West China School of Medicine, Sichuan University, Chengdu, China

**Keywords:** artificial intelligence, big five inventory, general attitudes toward AI scale, medical education, medical students

## Abstract

**Background:**

Artificial intelligence (AI) is reshaping healthcare from clinical care and diagnostics to operations and public health and has been accelerated by recent advances in large language models (LLMs), such as ChatGPT. Because healthcare directly concerns human life and well-being, the use of AI must be approached with care. As AI systems are ultimately designed, implemented, and trusted by people, identifying the factors that shape medical students’ attitudes toward AI is critical for safe and effective integration.

**Methods:**

In this study, attitudes toward AI were measured with the General Attitudes toward AI Scale (GAAIS), and personality traits were measured with the Big Five Inventory-2 (BFI-2). Along with demographic information, all these data were collected via an online platform from five-year clinical medicine students in Beijing, Shanghai, and Chengdu. Correlation and linear regression were conducted.

**Results:**

Analyses indicated that openness and agreeableness were associated with higher scores of positive attitudes toward AI, whereas conscientiousness was associated with lower scores of positive attitudes toward AI. For negative attitudes toward AI, openness and agreeableness were associated with its higher scores, whereas neuroticism was associated with its lower scores.

**Conclusion:**

These results suggest that students with greater openness and agreeableness not only view AI more favorably but are also more tolerant of its limitations. In contrast, those high in conscientiousness report fewer positive views, and those high in neuroticism are less tolerant of AI shortcomings. To our knowledge, this is the first study to examine the associations between personality traits and attitudes toward AI in medical students in China, highlighting the need for targeted educational interventions that reflect diverse personality profiles.

## Introduction

Artificial intelligence (AI) is transforming healthcare, permeating clinical care, diagnostics, operations, and public health, with the development of AI in recent years, especially the breakthrough of large language models (LLMs) such as the ChatGPT. AI can increase efficiency, accuracy, and equity across different aspects of healthcare systems, such as computer vision, which marks lesions on images; predictive analytics, which anticipate disease outcomes; robotic assistance in procedures; and LLM tools, which streamline documentation and patient communication (1, 2). Since AI has been rapidly adopted in healthcare systems and is gaining increasing importance, rigorous research and evaluation are necessary (3, 4).

As healthcare is a distinctive domain intimately tied to the protection of human life and well-being, the utilization of AI should be considered. In addition, medical students constitute the backbone of the future healthcare workforce; therefore, examining their attitudes toward AI is highly important for guiding its responsible integration into health services and education. Recent evidence suggests that generative AI has already entered medical students’ learning practices. In a survey of German medical students, 76.2% reported using ChatGPT for medical education, most commonly for summarizing information, literature research, and clarifying concepts, highlighting its perceived efficiency while also raising concerns about misinformation and trust (5). Similarly, among graduating medical students surveyed in the United Arab Emirates, reported usage was lower in medical students, with anticipating use for exam preparation and exploring new medical topics (6). In the United States, recent work has begun to characterize frequency and motivations for ChatGPT use among medical students, reflecting growing integration into learning, writing, and research workflows. The most frequently used tools are LLM tools, typically applied for study support, drafting, and information synthesis rather than direct clinical decision-making (5). Importantly, policies aligned with its use exist: The Association of American Medical Colleges (AAMC) has issued principles to guide AI use in medical education, emphasizing transparency, privacy protection, and human-centered oversight (7), while universities increasingly publish guidance and syllabus statements for AI usage (8). At the governance level, United Nations Educational, Scientific and Cultural Organization (UNESCO) has released global guidance for AI in education and research (9). Taken together, these surveys and policy statements indicate that AI use is already embedded in medical training, while concerns about reliability, trust, and responsible use remain; understanding students’ attitudes and their determinants is therefore important for curriculum planning and governance.

Studies have investigated attitudes toward AI among both healthcare professionals and medical students. For healthcare professionals, an integrative review synthesizing 42 studies highlighted barriers such as concerns about autonomy, workflow fit, legal responsibility, and job displacement, alongside facilitators including training, usable interfaces, and organizational readiness (10). In addition, for medical students, a meta-analysis estimated that approximately 65% of medical, dental, and nursing students view AI positively, with marked between-country variability (11). This heterogeneity implies that beyond exposure and training, individual-level determinants may meaningfully shape how students evaluate AI. To support comparable measurement, the General Attitudes toward AI Scale (GAAIS) provides a robust two-factor (positive/negative) structure and has been validated across settings (12). Beyond educational exposure and contextual factors, stable individual differences may also shape medical students’ attitudes toward AI. In this study, we focus on personality traits as a theoretically grounded candidate determinant.

The classic Big Five personality framework classifies personality into five broad domains: openness, conscientiousness, extraversion, agreeableness and neuroticism (OCEAN). Openness reflects curiosity and a preference for novelty; conscientiousness captures self-discipline, organization, and reliability; extraversion indexes energetic social engagement; agreeableness denotes a prosocial, cooperative, and compassionate stance; neuroticism reflects a tendency toward negative affect, stress reactivity, and emotional lability. These dimensions, supported by decades of lexical and psychometric research, are stable and predictive of social behavior, well-being, and occupational and health outcomes (13). To assess these traits efficiently and with facet-level precision, the Big Five Inventory-2 (BFI-2) provides a 60-item measure that yields five domain scores and fifteen facet scores, enhancing bandwidth, fidelity, and interpretability in empirical research and practice (14). These traits may be relevant to AI adoption because they relate to how individuals approach novelty, uncertainty, and perceived risk, which may influence receptivity to AI and tolerance of its limitations.

An expanding body of literature explores how personality shapes attitudes toward AI in healthcare across cultures. In Turkey, dentistry students’ views on AI vary by personality profile (15), and work from Saudi Arabia suggests that all Big Five traits except extraversion were associated with scores of attitudes toward AI (16). In South America, studies indicate that personality dispositions influence healthcare workers’ willingness to adopt AI, with patterns conditioned by cultural norms and system structures (17). Research in African contexts underscores context-specific barriers to and facilitators of AI-enabled care, reinforcing the need for culturally responsive implementation strategies (18). Across Asia, rapid AI adoption in health services has prompted investigations among nursing students and professionals; these investigations link personality traits to AI acceptance and often diverge from Western findings (19–21). Nevertheless, to our knowledge, no study has yet examined the associations between personality and attitudes toward AI among Chinese medical students. Cross-cultural evidence suggests that attitudes toward AI are heterogeneous and may depend on sociocultural and educational contexts, making it inappropriate to assume that findings from other settings generalize directly to Chinese medical students (11). Although recent studies have begun to describe AI attitudes among medical students in China, the evidence remains limited and rarely examines psychological determinants such as personality traits using standardized attitude measures (22).

Our study aimed to investigate the associations between Chinese medical students’ attitudes toward AI and personality traits. The demographic data, personality traits and attitudes toward AI were collected from online questionnaires. The relationships between personality traits and attitudes toward AI were investigated through correlation analysis, and the personality traits that were associated with attitudes toward AI were investigated through linear regression. This study extends the emerging literature on psychological determinants of AI attitudes by examining whether Big Five traits are associated with attitudes toward AI in Chinese medical students, a context where evidence remains limited and cross-cultural patterns are heterogeneous. The findings also have practical value for medical education, as identifying trait-linked profiles of receptivity and caution can inform curriculum design and risk-aware training strategies for responsible AI use.

## Materials and methods

### Participants

A cross-sectional, web-based survey was conducted. Participants were recruited via convenience sampling from three public universities in Beijing, Shanghai, and Chengdu: Peking University Health Science Center; the School of Medicine, Shanghai Jiao Tong University; and the West China School of Clinical Medicine, Sichuan University from July to October, 2025. Eligible respondents were undergraduate medical students enrolled in a five-year clinical medicine program (the most common undergraduate track in China) at any year level who consented to complete the survey. The exclusion criteria were postgraduate status; current mental health difficulties or ongoing psychological treatment; and enrollment in self-improvement or professional development courses. To ensure that the target population completed the survey, eligibility was verified via self-report using mandatory screening items based on the inclusion and exclusion criteria.

The sample size was estimated with PASS (version 21.0.3, 2021) for a linear multiple regression framework. Using nine predictors (four demographic variables and the Big Five personality traits), an anticipated medium effect (*f*^2^ = 0.15) (23), *α* = 0.01, and 95% power, the minimum required *N* was 228. Considering an expected attrition rate of 12% for a web-based survey, the target sample size was inflated to 260 (24). Two attention check questions (If you could select “Strongly agree,” I would greatly appreciate it) were involved in the questionnaire (one in the GAAIS part and the other in the BFI-2 part). Participants selecting “Strongly agree” in both of the 2 attention check questions were included in our study. Ultimately, 253 medical students completed the web-based questionnaire after excluding 5 participants who did not pass the attention check, and were included in the final analysis, exceeding the minimum required sample size.

### Demographic data

The demographic characteristics recorded included age, gender, marital status, and educational attainment.

### GAAIS

The General Attitudes toward Artificial Intelligence Scale (GAAIS), developed by Schepman and Rodway (12), assesses overall dispositions toward AI. The original instrument contains 20 items across two subscales: positive attitudes (12 items) and negative attitudes (8 items). Subscales are scored separately, with higher values indicating stronger endorsement of positive or negative views. In the initial validation, internal consistency was high (*α* = 0.88 for the positive subscale; α = 0.83 for the negative subscale). To accommodate linguistic and cultural contexts, Huang et al. created a Chinese adaptation comprising 15 items (8 positive, 7 negative) (25). Positive items are rated on a five-point Likert scale (1 = strongly disagree to 5 = strongly agree); negative items are reverse-coded (1 = strongly agree to 5 = strongly disagree). Subscale scores are calculated as the means of their respective items. Higher scores on either subscale reflect more favorable attitudes toward AI; for the negative subscale, a higher mean denotes greater tolerance of AI limitations. The Chinese version used in our study showed good reliability, with Cronbach’s *α* ranging from 0.833–0.875, and convergent validity was supported by systematic correlations with measures of technology readiness and personality (25).

### BFI-2

The Big Five Inventory (BFI), developed by Benet-Martínez and John, assesses five broad personality domains with 44 items: extraversion (8), agreeableness (9), conscientiousness (9), neuroticism (8), and openness (10) (26). The revised BFI-2 expands the instrument to 60 items, assigning 12 items to each domain (14). Zhang et al. translated BFI-2 into Chinese, which was applied in our study, and demonstrated its good reliability, structural validity, convergent/discriminant validity, and criterion-related validity at the domain level (27).

### Data collection and ethics

Data were gathered via a questionnaire hosted on WenJuanXing, a widely used online survey platform. The link was disseminated mainly via instant messaging tools in China, such as WeChat and QQ. The opening page provided an invitation describing the study objectives and intended participant group. Participation was voluntary, with assurances of confidentiality. Electronic informed consent was obtained; respondents had to click “I agree” before proceeding. To maintain data quality, all the items were compulsory, and the system prevented the submission of incomplete forms. The survey was anonymous and emphasized that there were no right or wrong answers to reduce social desirability and evaluation apprehension. The study protocol was approved by the Ethics Committee of West China Hospital, Sichuan University (Approval No. 2024–0371).

### Statistical analyses

All analyses were conducted in SPSS 25.0. Participant demographics and study variables were summarized using frequencies and percentages, as well as means and standard deviations. Procedures were implemented to evaluate the measures’ validity and reliability. The common method variance (CMV) was assessed using Harman’s single-factor test (unrotated principal component factor analysis of all GAAIS and BFI-2 items). Between-group differences by demographic factors were tested with independent-samples t tests and one-way ANOVA. Associations between personality dimensions and attitudes toward AI were examined with Pearson correlation coefficients. Finally, stepwise multiple linear regressions were fitted separately for the positive and negative attitude scores.

## Results

### Participant demographics

The participants were approximately evenly distributed by age, gender, and year of study; all the participants were unmarried, reflecting the rarity of marriage among Chinese undergraduates. No statistically significant demographic differences were observed in medical students’ attitudes toward AI (Table 1).

**Table 1:** Participant demographics and between-group differences in attitudes toward AI (*N* = 253).

Category		*N*	%	Positive		Negative	
				Mean (SD)	*t*/*F* (*p* value)	Mean (SD)	*t*/*F* (*p* value)
Age	<20	106	41.9	2.62 (1.01)	*t* = 1.50 (0.14)	3.15 (1.06)	*t* = −1.40 (0.162)
	≥20	147	58.1	2.42 (1.07)		3.36 (1.20)	
Gender	Male	116	45.8	2.63 (1.01)	*t* = 1.84 (0.066)	3.41 (1.18)	*t* = 1.73 (0.085)
	Female	137	54.2	2.39 (1.07)		3.16 (1.11)	
Marital status	Married	0	0.0	NA	NA	NA	NA
	Unmarried	253	100.0	2.50 (1.05)		3.27 (1.15)	
Educational level	1st year	39	15.4	2.39 (0.94)	*F* = 1.87 (0.117)	3.13 (1.09)	*F* = 1.087 (0.363)
	2nd year	68	26.9	2.70 (1.05)		3.24 (1.02)	
	3rd year	54	21.3	2.22 (1.09)		3.48 (1.24)	
	4th year	59	23.3	2.61 (1.03)		3.36 (1.18)	
	5th year	33	13.0	2.50 (1.07)		3.02 (1.24)	

### Descriptive statistics and reliability

Table 2 shows that negative attitudes (*M* = 3.27, SD = 1.15) scored more than positive attitudes did (*M* = 2.50, SD = 1.05). The mean scores for personality traits were as follows: openness (*M* = 3.53, SD = 0.62), conscientiousness (*M* = 3.21, SD = 0.44), extraversion (*M* = 3.18, SD = 0.43), agreeableness (*M* = 3.46, SD = 0.63), and neuroticism (*M* = 2.90, SD = 0.39). Harman’s single-factor test indicated that the first factor accounted for 11.36% of the total variance, suggesting that CMV was unlikely to substantially bias the observed associations.

**Table 2:** Descriptive statistics and reliability (*N* = 253).

Variable	Mean (SD)	Cronbach’s α
Attitude toward AI		
Positive attitude	2.50 (1.05)	0.85
Negative attitude	3.27 (1.15)	0.88
Personality traits		
Openness	3.53 (0.62)	0.84
Conscientiousness	3.21 (0.44)	0.89
Extraversion	3.18 (0.43)	0.76
Agreeableness	3.46 (0.63)	0.85
Neuroticism	2.90 (0.39)	0.76

Reliability was evaluated via Cronbach’s *α*, and the α coefficients ranged from 0.76–0.89 across all factors, exceeding the conventional 0.70 benchmark and indicating strong internal consistency.

### Correlation analysis between attitudes toward AI and personality traits

**Figure 1:**
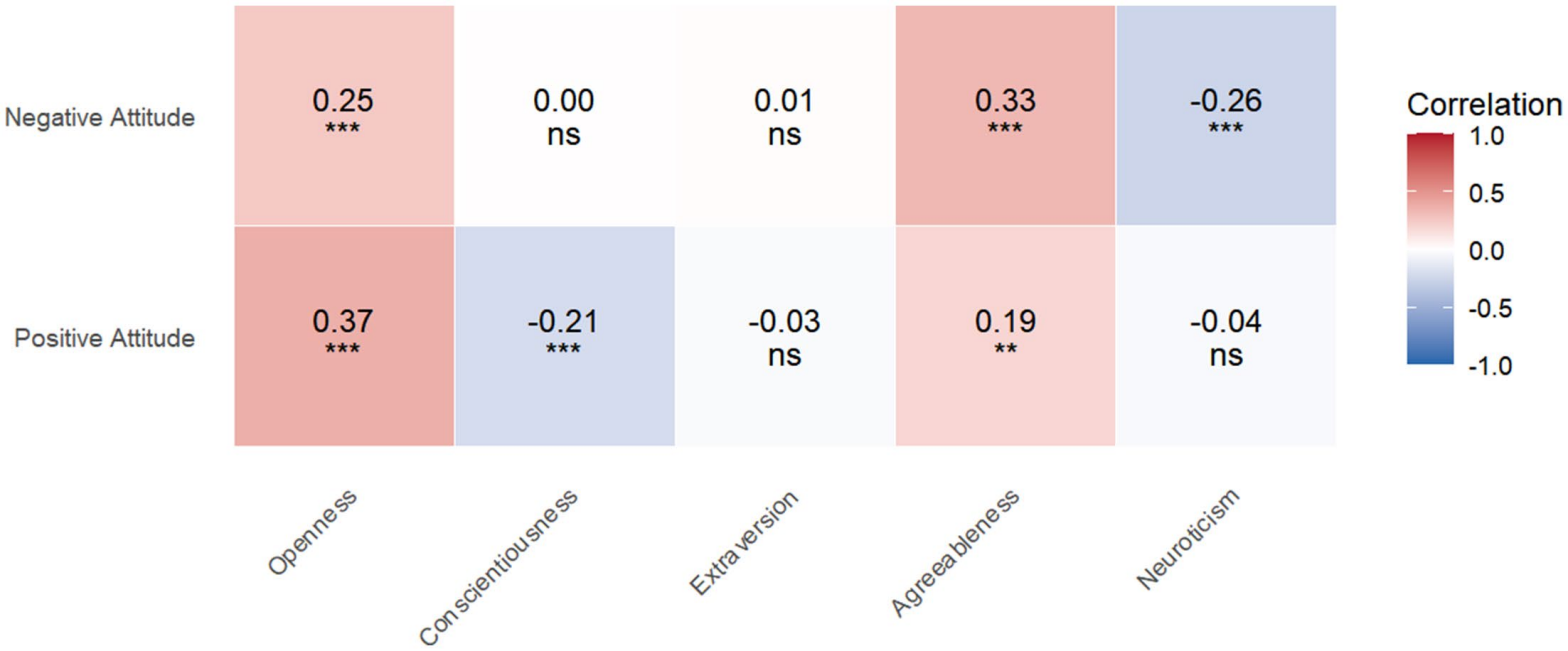
The heatmap showed the correlation between attitudes toward AI and personality traits. Redder color indicates stronger positive correlations while bluer color indicates stronger negative correlations. *p < 0.05, **p < 0.01, ***p < 0.001.

Table 3 reports the Pearson correlation results. Positive attitudes toward AI were significantly associated with greater openness (*r* = 0.37, *p* < 0.001) and agreeableness (*r* = 0.19, *p* = 0.003) and with lower conscientiousness (*r* = −0.21, *p* = 0.001). For negative attitudes, openness (*r* = 0.25, *p* < 0.001) and agreeableness (*r* = 0.33, *p* < 0.001) had positive associations, whereas neuroticism had a negative association (*r* = −0.26, *p* < 0.001). The correlation coefficient and *p* value were visualized in Figure 1.

**Table 3:** The correlations between attitudes toward AI and personality traits (*N* = 253).

Personality traits	Attitudes toward AI	
	Positive	Negative
Openness		
Correlation coefficient *r*	0.37	0.25
*p* value	<0.001	<0.001
Conscientiousness		
Correlation coefficient *r*	−0.21	0.004
*p* value	0.001	0.954
Extraversion		
correlation coefficient r	−0.031	0.013
*p* value	0.628	0.835
Agreeableness		
correlation coefficient r	0.19	0.33
*p* value	0.003	<0.001
Neuroticism		
correlation coefficient r	−0.038	−0.26
*p* value	0.552	<0.001

### Linear regression analysis for predictors of attitudes toward AI

Hierarchical multiple linear regression predicting attitudes toward AI was conducted: demographic covariates (age, gender, and educational level dummies) were forced into Block 1 using the Enter method to control potential confounding, and the personality traits that were significant in the correlation analyses were selected in Block 2 using stepwise procedures.

**Figure 2:**
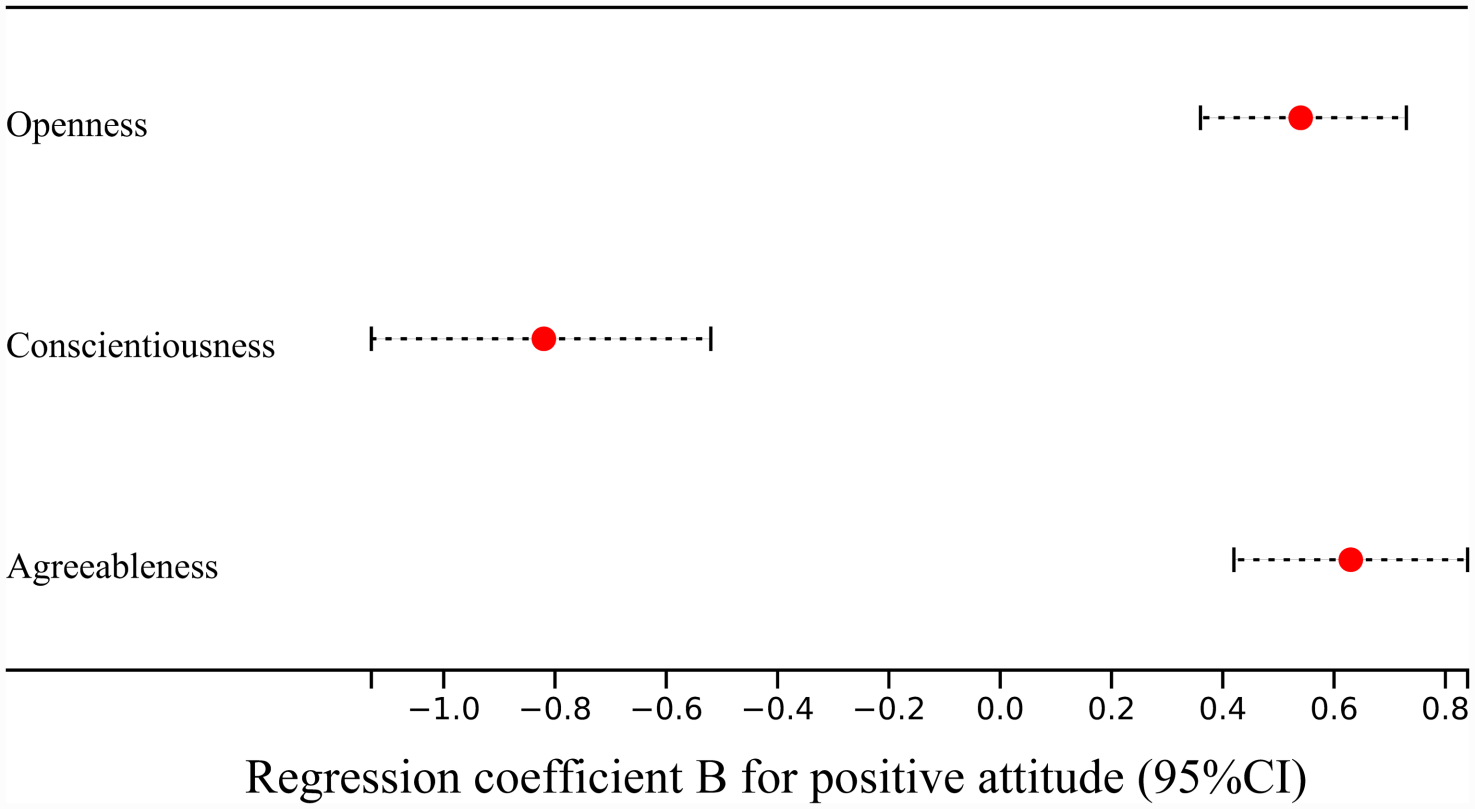
The forest plot showed the regression coefficient B of personality traits in the regression model for positive attitude.

In hierarchical regression predicting positive attitudes toward AI, Block 1 (age, gender, and educational level) was not significant (adjusted R^2^ = 0.002, *p* = 0.32). In Block 2, stepwise selection retained openness, agreeableness, and conscientiousness as significant predictors. Adding openness significantly improved model fit (ΔR^2^ = 0.1, *p* < 0.001), followed by agreeableness (ΔR^2^ = 0.04, *p* = 0.001) and conscientiousness (ΔR^2^ = 0.084, *p* < 0.001). The final model explained 24.7% of the variance in positive attitudes (adjusted R^2^ = 0.247, *p* < 0.001). In the final model, openness (*β* = 0.32, *p* < 0.001) and agreeableness (*β* = 0.38, *p* < 0.001) were positively associated with positive attitudes, whereas conscientiousness was negatively associated (*β* = −0.35, *p* < 0.001) (Table 4; Figure 2). Age, gender, and educational level were not significant. Collinearity diagnostics were conducted, the tolerance values ranged from 0.70–0.95, with VIFs ranging from 1.05–1.43, well above/below the conventional 0.10- and 3.0 cutoffs, respectively, and no multicollinearity issues were found for the positive-attitude models. Model-building details for Models 1–3 are provided in Supplementary Table S1.

**Table 4:** Final model for stepwise multiple linear regression results identifying predictors of medical students’ positive attitudes toward AI.

Predictors	B (95% CI)	*β*	*t*	*p* value
Age	0.021 (−0.25, 0.29)	0.035	0.15	0.88
Gender	0.068 (−0.16, 0.30)	0.032	0.58	0.56
Educational level	−0.035 (−0.41, 0.34)	−0.043	−0.19	0.85
Openness	0.54 (0.36, 0.73)	0.32	5.71	<0.001
Conscientiousness	−0.82 (−1.13, −0.52)	−0.35	−5.86	<0.001
Agreeableness	0.63 (0.42, 0.84)	0.38	5.30	<0.001

B, unstandardized regression coefficient; CI, confidence interval; *t*, *t*-statistics.

**Figure 3:**
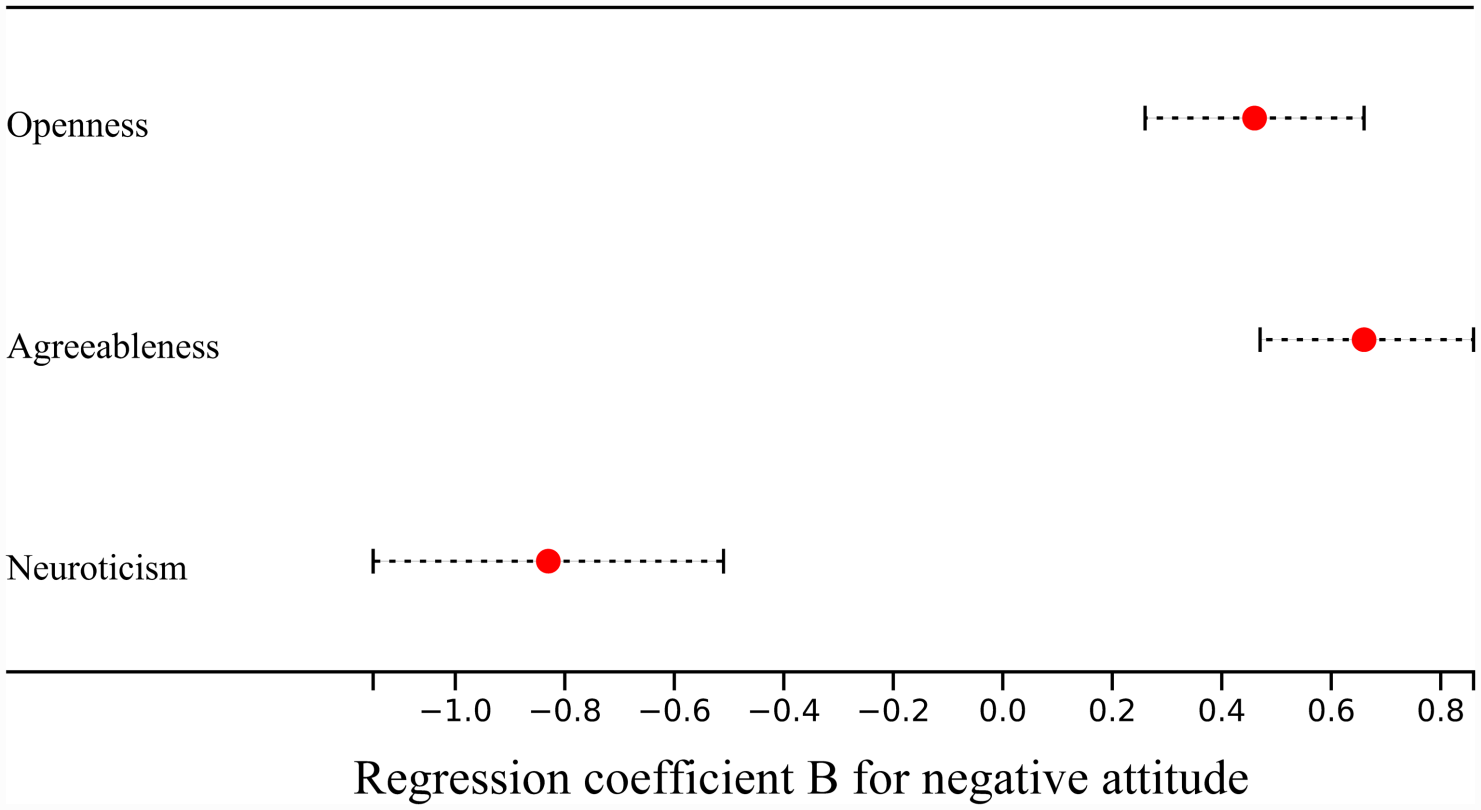
The forest plot showed the regression coefficient B of personality traits in the regression model for negative attitude.

In hierarchical regression predicting negative attitudes toward AI, Block 1 (age, gender, and educational level) showed a small amount of explained variance and did not reach statistical significance (adjusted R^2^ = 0.016, *p* = 0.074). In Block 2, stepwise selection retained agreeableness, neuroticism, and openness as significant predictors. Adding agreeableness significantly improved model fit (ΔR^2^ = 0.110, *p* < 0.001), followed by neuroticism (ΔR^2^ = 0.077, *p* < 0.001) and openness (ΔR^2^ = 0.059, *p* < 0.001). The final model explained 25.7% of the variance in negative attitudes (adjusted R^2^ = 0.257, *p* < 0.001). In the final model, agreeableness (*β* = 0.36, *p* < 0.001) and openness (*β* = 0.25, *p* < 0.001) were positively associated with negative attitudes, whereas neuroticism was negatively associated (*β* = −0.28, *p* < 0.001). Educational level showed a small negative association (*β* = −0.46, *p* = 0.048), while age and gender were not significant in the final model (Table 5; Figure 3). Collinearity diagnostics were conducted, the tolerance values ranged from 0.98–0.99, with VIFs ranging from 1.015–1.024, well above/below the conventional 0.10- and 3.0 cutoffs, respectively, and no multicollinearity issues were found for the negative-attitude models. Detailed coefficients for Models 1–3 are provided in Supplementary Table S2.

**Table 5:** Stepwise multiple linear regression results identifying predictors of medical students’ negative attitudes toward AI.

Predictors	B (95% CI)	*β*	*t*	*p* value
Age	0.27 (−0.029, 0.57)	0.41	1.78	0.077
Gender	0.17 (−0.077, 0.42)	0.075	1.36	0.18
Educational level	−0.41 (−0.82, −0.003)	−0.46	−1.98	0.048
Openness	0.46 (0.26, 0.66)	0.25	4.49	<0.001
Agreeableness	0.66 (0.47, 0.86)	0.36	6.63	<0.001
Neuroticism	−0.83 (−1.15, −0.51)	−0.28	−5.08	<0.001

B, unstandardized regression coefficient; CI, confidence interval; *t*, *t*-statistics.

## Discussion

This study investigated how personality traits shape Chinese medical students’ attitudes toward AI. Although prior work has assessed healthcare professionals’ views, evidence focused on medical students in China is sparse. Drawing on the BFI framework, we examine whether specific traits were associated with variation in attitudes toward AI, addressing this gap. Overall, attitudes toward AI among Chinese medical students appeared less differentiated by demographics and more closely related to personality. The findings suggested that openness and agreeableness were linked to greater receptivity toward AI, while conscientiousness and neuroticism were linked to more reserved evaluations, highlighting the value of tailoring AI education to individual differences. Our analyses revealed that demographic characteristics appeared to play a limited role in shaping attitudes toward AI. This aligns with prior evidence that the cultural context shapes technology perceptions and that a shared cultural background may yield similar views. This interpretation is consistent with prior work suggesting that cultural context and shared norms can strongly influence how new technologies are understood and evaluated (17, 28, 29). In contrast, individual psychological differences, particularly personality traits, may become more salient in explaining why some students are more receptive to AI whereas others remain more cautious.

Consistent with prior research, individuals high in openness are typically curious, innovation oriented, and receptive to new technologies. This disposition may make students more willing to experiment with AI, view it as a tool for learning and improvement, and remain more comfortable with its inevitable imperfections in clinical use (30, 31). Similarly, Salem et al. reported that openness was associated with higher scores of positive attitudes toward AI among nursing students (16). Taken together, this pattern supports the broader view that openness facilitates constructive engagement with emerging technologies during healthcare training.

Moreover, agreeableness may be linked to more favorableness toward AI and greater tolerance of its shortcomings. Because agreeableness reflects compassion and cooperativeness, students who score higher on this trait may adopt AI more readily and show greater tolerance of its limitations than their less agreeable peers do. Nonetheless, this pattern differs from that of Salem et al. and other studies reporting no significant link between agreeableness and positive attitudes toward AI. The cultural context may help explain this discrepancy. East Asian students, shaped by collectivist norms from early childhood, tend to value cooperation, which may promote greater tolerance (32).

Conscientiousness may be associated with more reserved evaluations of AI among medical students, particularly when students consider the risks and uncertainties of clinical use. This pattern may reflect greater meticulousness and adherence to protocols, which in turn fosters heightened cautiousness of AI limitations. However, evidence elsewhere is mixed. Kaya et al. reported no predictive effect of conscientiousness on either positive or negative attitudes (30), whereas other studies reported a weak but significant positive association with negative attitudes (33, 34). These discrepancies underscore the need for further investigation.

Furthermore, neuroticism may reduce students’ tolerance for AI’s shortcomings by amplifying anxiety, skepticism, and perceived threat in response to AI-related uncertainty. This finding is consistent with prior evidence that highly neurotic individuals are more prone to anxiety, skepticism, and fear regarding AI risks and implications (31). They may perceive AI as a threat rather than a tool, and the prospect of machines emulating human capabilities and displacing human tasks can amplify the insecurity and job-loss concerns of these participants, thereby reinforcing unfavorable attitudes (35, 36).

However, extraversion appeared to play a limited role in shaping medical students’ attitudes toward AI, which is consistent with prior reports showing no significant associations (16, 30, 31, 33). One possible explanation is that extraversion may be less directly relevant to evaluations of AI as a clinical tool, with its influence depending on more interpersonal or social-use contexts. In addition, within an East Asian collectivist cultural background, where social harmony and solidarity are emphasized over individual expressiveness, differences related to extraversion may be less salient in shaping attitudes toward AI.

This study has several limitations. First, the sample was restricted to students in five-year clinical medicine programs, the most common undergraduate track in China, so the findings may not be generalizable to 7- or 8-year programs or to dental students, and future work should include these groups. Second, the cross-sectional design limits causal inference, and longitudinal studies are needed to track how personality relates to attitudes over time and across training and clinical contexts. Third, all variables were collected via self-report, which may be subject to recall and social desirability bias. CMV is a potential concern given the self-report design. Nevertheless, Harman’s single-factor test provided some reassurance (the first factor explained 11.36% of total variance), although CMV cannot be fully excluded. In addition, although we used Chinese versions with published psychometric support, we did not test cross-cultural measurement invariance of the GAAIS or BFI-2; therefore, the present findings should not be directly generalized to other cultural contexts or used for cross-country comparisons without further equivalence testing. Furthermore, this study used a web-based convenience sample recruited via social media, which may introduce selection and coverage bias. Students who were more interested in AI or more engaged online may have been more likely to participate, limiting representativeness and generalizability. Although we recruited participants from three universities in different regions, the sample may not reflect all Chinese medical students. Future studies should consider probability-based or stratified sampling and mixed-mode (online/offline) recruitment.

### Implications

To our knowledge, this is the first study to examine the associations between personality traits and attitudes toward AI among Chinese medical students. Taken together, the findings suggest that individual differences may shape whether students approach AI with curiosity and openness to collaboration, or with greater caution when considering uncertainty and potential risks (37). From a curricular perspective, AI content is likely to be most effective when it is integrated longitudinally across the existing program (introduced early and revisited in clinically anchored contexts), rather than delivered as a single, stand-alone module (38). This integration can be operationalized through specialty-relevant cases and structured critical-appraisal activities, and consolidated using simulation or OSCE-style tasks that require safe human-AI collaboration, documentation of AI-assisted reasoning, and communication with patients about uncertainty, ethics, and governance. Pedagogically, tailoring can be modest but practical: students who are more receptive to AI may benefit from team-based projects (e.g., workflow mapping, structured evaluation of a published clinical AI tool, or guided use-case development), whereas students who are more cautious or less tolerant of AI shortcomings may benefit from stepwise exposure, verification checklists, error-analysis exercises, and supported debriefing to build calibrated trust without overreliance (37). In particular, for groups with lower tolerance, teaching should explicitly emphasize competencies repeatedly highlighted in recent competency frameworks and curriculum guidance-recognizing limitations and failure modes, uncertainty calibration and verification, bias/fairness awareness, ethical and legal accountability, and monitoring AI use in real clinical workflows (39).

## Conclusion

In our study, the data on attitudes toward AI were collected through the GAAIS, and personality trait data were collected through the BFI-2, both via an online system. The correlations between attitudes toward AI and personality traits were explored. The traits that were significantly correlated with positive and negative attitudes were subsequently selected via linear regression. In the regression model, we found that for positive attitudes toward AI, openness and agreeableness were associated with its higher scores, whereas conscientiousness was associated with its lower scores. For negative attitudes toward AI, openness and agreeableness were associated with its higher scores, whereas neuroticism was associated with its lower scores. These findings suggested that students with high openness and agreeableness not only had more positive attitudes toward AI but were also more tolerant of the shortcomings of AI. Students with high conscientiousness had fewer positive attitudes toward AI, and students with high neuroticism were not tolerant of the shortcomings of AI. Our study is the first to investigate the relationship between attitudes toward AI and personality traits in medical students, suggesting the need for targeted educational interventions that consider the diverse personality traits of medical students.

## Data Availability

The raw data supporting the conclusions of this article will be made available by the authors, without undue reservation.
