# Supplementary material for: The relationship between medical students’ attitudes toward artificial intelligence and their personality traits: a multicenter study in China
Source: Front Public Health. 2026 Feb 4;14:1749279. doi: 10.3389/fpubh.2026.1749279 (PMC12913472; doi:10.3389/fpubh.2026.1749279)
Supplement: Supplementary file 1 [file Table_1.docx]

**Table S1. Model 1 to Model 3 for stepwise multiple linear regression results identifying predictors of medical students’ positive attitudes toward AI**

| **Predictors** | **B (95% CI)** | **β** | **t** | **p value** | **B (95% CI)** | **β** | **t** | **p value** | **B (95% CI)** | **β** | **t** | **p value** |
| --- | --- | --- | --- | --- | --- | --- | --- | --- | --- | --- | --- | --- |
|  | *Model 1* | | | | *Model 2* | | | | *Model 3* | | | |
| Age | 0.059 (-0.26,0.37) | 0.099 | 0.37 | 0.71 | 0.004 (-0.29,0.30) | 0.007 | 0.028 | 0.98 | 0.016 (-0.27,0.30) | 0.027 | 0.11 | 0.91 |
| Gender | 0.238 (-0.23, 0.5) | 0.11 | 1.80 | 0.074 | 0.15 (-0.1,0.39) | 0.07 | 1.17 | 0.24 | 0.13 (-0.11,0.37) | 0.062 | 1.07 | 0.29 |
| Educational level | -0.08 (-0.51,0.35) | -0.098 | -0.37 | 0.71 | -0.016 (-0.42, 0.38) | -0.2 | -0.08 | 0.94 | -0.046 (-0.44,0.35) | -0.056 | -0.23 | 0.82 |
| Openness | - | - | - | - | 0.61 (0.41,0.81) | 0.36 | 6.07 | <0.001 | 0.628 (0.43,0.82) | 0.37 | 6.36 | <0.001 |
| Agreeableness | - | - | - | - | - | - | - | - | 0.335 (0.15,0.53) | 0.20 | 3.47 | 0.001 |
| Conscientiousness | - | - | - | - | - | - | - | - | - | - | - | - |
