# Supplementary material for: The relationship between medical students’ attitudes toward artificial intelligence and their personality traits: a multicenter study in China
Source: Front Public Health. 2026 Feb 4;14:1749279. doi: 10.3389/fpubh.2026.1749279 (PMC12913472; doi:10.3389/fpubh.2026.1749279)
Supplement: Supplementary file 2 [file Table_2.docx]

**Table S2. Model 1 to Model 3 for stepwise multiple linear regression results identifying predictors of medical students’ negative attitudes toward AI**

| **Predictors** | **B (95% CI)** | **β** | **t** | **p value** | **B (95% CI)** | **β** | **t** | **p value** | **B (95% CI)** | **β** | **t** | **p value** |
| --- | --- | --- | --- | --- | --- | --- | --- | --- | --- | --- | --- | --- |
|  | *Model 1* | | | | *Model 2* | | | | *Model 3* | | | |
| Age | 0.35 (0.006, 0.69) | 0.53 | 2.01 | 0.046 | 0.37 (0.05,0.70) | 0.57 | 2.28 | 0.024 | 0.31 (0.00,0.618) | 0.47 | 1.97 | 0.050 |
| Gender | 0.22 (-0.061,0.51) | 0.097 | 1.55 | 0.12 | 0.20 (-0.07, 0.47) | 0.086 | 1.46 | 1.457 | 0.24 (-0.015,0.50) | 0.11 | 1.86 | 0.065 |
| Educational level | -0.46 (-0.92,0.005) | 0.51 | -1.95 | 0.053 | -0.52 (-0.96, -0.077) | -0.58 | -2.32 | 0.021 | -0.46 (-0.88, -0.033) | -0.51 | -2.13 | 0.035 |
| Agreeableness | - | - | - | - | 0.61 (0.40, 0.82) | 0.33 | 5.63 | <0.001 | 0.64 (0.44, 0.85) | 0.35 | 6.19 | <0.001 |
| Neuroticism | - | - | - | - | - | - | - | - | -0.83 (-1.17, 0.50) | -0.28 | -4.93 | <0.001 |
| Openness | - | - | - | - | - | - | - | - | - | - | - | - |
